# The biological role of MutT in the pathogenesis of the zoonotic pathogen *Streptococcus suis* serotype 2

**DOI:** 10.1080/21505594.2021.1936770

**Published:** 2021-06-02

**Authors:** Quan Li, Xia Fei, Yuhang Zhang, Genglin Guo, Huoying Shi, Wei Zhang

**Affiliations:** a College of Veterinary Medicine, Yangzhou University, Yangzhou, China; b Jiangsu Co-innovation Center for the Prevention and Control of Important Animal Infectious Diseases and Zoonoses, Yangzhou, China; c MOE Joint International Research Laboratory of Animal Health and Food Safety, Key Lab of Animal Bacteriology, Ministry of Agriculture, OIE Reference Lab for Swine Streptococcosis, College of Veterinary Medicine, Nanjing Agricultural University, Nanjing, China

**Keywords:** *Streptococcus suis*, MutT, pathogenesis, virulence

## Abstract

*Streptococcus suis* (*S. suis*) is an important rising pathogen that causes serious diseases in humans and pigs. Although some putative virulence factors of *S. suis* have been identified, its pathogenic mechanisms are largely unclear. Here, we identified a putative virulence-associated factor MutT, which is unique to *S. suis* serotype 2 (SS2) virulent strains. To investigate the biological roles of MutT in the SS2 virulent strain ZY05719, the *mutT* knockout mutant (Δ*mutT*) was generated and used to explore the phenotypic and virulent variations between the parental and Δ*mutT* strains. We found that the *mutT* mutation significantly inhibited cell growth ability, shortened the chain length, and displayed a high susceptibility to H_2_O_2_-induced oxidative stress. Moreover, this study revealed that MutT induced the adhesion and invasion of SS2 to host cells. Deletion of *mutT* increased microbial clearance in host tissues of the infected mice. Sequence alignment results suggested that *mutT* was encoded in a strain-specific manner, in which the detection was strongly linked to bacterial pathogenicity. In both zebrafish and mice infection models, the virulence of Δ*mutT* was largely reduced compared with that of ZY05719. Overall, this study provides compelling evidence that MutT is indispensable for the virulence of SS2 and highlights the biological role of MutT in bacteria pathogenesis during infection.

## Introduction

*Streptococcus suis* (*S. suis*) is an emerging zoonotic pathogen in the swine, which causes diverse infections in pigs like septicemia, meningitis, endocarditis, and arthritis [1]. Seriously, it may result in septicemia, meningitis, or even sudden death in humans [2]. *S. suis* has spread worldwide and caused enormous economic losses in the swine industry in recent years because of its high morbidity and mortality [1]. Categorized by capsular antigens, there are 35 serotypes (types 1 to 34, and 1/2) of *S. suis*, in which serotype 2 (SS2) is the most frequently and virulent isolated from sick piglets. Historically, SS2 has caused two large outbreaks of human infection in 1998 and 2005, which has aroused public attention in China [3]. However, the molecular pathogenesis of *S. suis*-induced infectious disease is still limited and fragmentary [4,5].

To date, over 100 putative virulence factors have been identified associated with *S. suis* virulence. These putative virulence factors can be classified into three categories based on their biological functions in the cycles of bacterial life: 1) Surface and secreted proteins like capsular polysaccharide (CPS) [6,7], suilysin (SLY) [8–10], extracellular protein factor (EF) [11], and muramidase-released protein (MRP) [11–14]; 2) Enzymes and proteases like adenosine synthase [15], enolase [16–18], glutamine synthetase (GlnA) [19], and sialic acid synthase (NeuB) [20]; 3) Transcription factors and regulatory systems like CodY [21], AdcR [22], catabolite control protein A (CcpA) [23,24] and three TCS systems, including SalK-SalR [25], NisK-NisR [26], and VirR-VirS [27]. However, the virulence of *S. suis* strains is highly diverse among different serotypes. Because of the presence of one or more virulence factors, whether a *S. suis* strain is virulent or avirulent is difficult to be defined [4]. The lack of a suitable virulent marker hinders the study of pathogenic mechanism of *S. suis* [5,28]. Therefore, the exact functions of these virulence factors remain to be further evaluated, which are of significance in dissecting the pathogenesis of *S. suis*.

Comparative genomic analysis is an efficient approach to identify genes responsible for the pathogenesis and virulence of bacteria. Several pathogenicity islands and genes, including a type IV-like secretion system [29], 89 K pathogenicity island [30], a type VII secretion system putative substrate *esxA* [31], and *pnuC* [32], have already been identified by this method. Our previous study revealed that the gene *mutT* (ZY05719_09350, the functional annotation in ZY05719 is DNA mismatch repair protein MutT) is unique to SS2 virulent strains by comparing the core genomes of virulent and avirulent SS2 strains [33], suggesting a potential correlation between MutT and *S. suis* virulence. However, the biologic functions of MutT in SS2 pathogenicity remain unknown. This study aims to characterize the biological role of MutT in SS2 pathogenicity and its function in bacteria virulence.

## Materials and methods

### Cell lines, bacterial strains, plasmids, and culture conditions

ZY05719, verified as a SS2 sequence type 7 virulent strain, was isolated from a diseased pig. The pSET4s plasmid was used for gene knockout, and the pMD19-T vector (Takara) was used to clone PCR fragments. Plasmids and bacterial strains applied in this study were listed in Table 1. The human brain microvascular endothelial cell line (HBMEC) and the human laryngeal epithelial cell line (HEp-2) were cultivated in DMEM (Gibco, Invitrogen) supplemented with 10% fetal bovine serum (FBS, Gibco) at 37°C. SS2 strains were cultured in a Todd Hewitt Broth (THB, Becton-Dicksinson) at 37°C. 100 µg/ml gentamicin and 5 µg/ml penicillin G were used to kill surface adhered and extracellular bacteria. Spectinomycin (50 µg/ml for *E. coli* and Spc, 100 µg/ml for SS2) was applied to screen transformants when required.

**Table 1.** Bacterial strains, plasmids, and primers used in this study

Strains, plasmid, or primer	Characteristics ^a^ or sequences (5ʹ–3ʹ)^b^	Sources or function
Strains		
ZY05719	Isolated from a diseased pig in Sichuan Province in China in 2005	Stored in our lab
*∆mutT*	Isogenic *mutT* mutant of strain ZY05719	This study
Plasmids		
PMD19-T	Cloning vector; Amp^R^	TaKaRa
pSET4s	*S. suis* thermosensitive suicide vector; Spc^R^	
*mutT*-4s	A recombinant vector with the background of pSET4s, designed to knockout *mutT*; Spc^R^	This study
Primers		
*mutT-*A	CCC**AAGCTT**GCAATCATTGCGGGACTATTT	Upstream flanking regions of *mutT*
*mutT-*B	CCTAGCCATTTCGAGCCACCT	
*mutT-*C	AGGTGGCTCGAAATGGCTAGGAGTTCATAAAAAGATAAAGAA	Downstream flanking regions of *mutT*
*mutT-*D	TCC**CCCGGG**GGTTGGAACAATACCGACCTG	
*mutT-*E	GGTATAAAAAGCAGGACGCTC	Internal regions of *mutT*
*mutT-*F	CTGCAGGAGATTGAGAAAGGT	
*mutT-*X	CATGTCTGCTATCGCAAATAG	External regions of *mutT*
*mutT-*Y	GAAATGGGAGCGGGACTGGTC	

^a^Amp^R^, Ampicillin resistance; Spc^R^, spectinomycin resistance; ^b^ Underlined nucleotides denote enzyme restriction sites.

### Ethics statement

BALB/c mice were provided by the Comparative Medicine Center of Yangzhou University. Animal experiments performed at Nanjing Agricultural University were approved by the Jiangsu Administrative Committee for Laboratory Animals (license number SCXK (SU) 2017–0007). In accordance with international laws, the procedures were conformed to the guidelines of Jiangsu Laboratory Animal Welfare and Ethics.

### Construction of the mutT mutant strain

The *mutT* deletion mutant (Δ*mutT*) was constructed in the wild-type strain ZY05719 using the thermosensitive suicide vector pSET4s. Primers for the amplification of the upstream (A/B) and downstream (C/D) flanking regions of the target gene *mutT* were listed in Table 1. The upstream and downstream fragments were fused as intact fragments by overlapping PCR, and they were cloned into the suicide vector pSET4s. Mutations were then introduced into ZY05719 using the suicide plasmid by electroporation as previously reported [32,34]. We screened the single-crossover mutants at 37°C by culturing the bacteria with Spc, and obtained the double-crossover mutants at 28°C by repeatedly passaging on Todd-Hewitt agar without Spc. The resulting mutant strain was confirmed by PCR using three primer pairs of external (X/Y), flanking (A/D) and internal (E/F) regions, as well as sequencing (A/D).

### Transmission electron microscopy (TEM)

TEM was performed to evaluate the effects of deletion of *mutT* on the morphology of ZY05719 as previously reported [32]. Briefly, ZY05719 and Δ*mutT* were harvested at OD_600_ of 0.6 by centrifugation and fixed in 5% glutaraldehyde at 25°C for 2 h. Then the strains were treated with 2% osmium tetroxide. We dehydrated the samples in a serial dilution of acetone washes and embedded them in epoxy resin. After fixation, the sections were post-stained with lead citrate and uranyl acetate, and observed using an H-7650 TEM (Hitachi, Tokyo, Japan). The average capsule thicknesses was measured in thirty randomly selected bacteria from ZY05719 or Δ*mutT* strain using Image J software, and analyzed using GraphPad.

### Bacterial growth curve assays

The SS2 strains ZY05719 and Δ*mutT* were cultured to an OD_600_ of 0.6 in THB at 37°C and inoculated into 50 ml of fresh THB (1:100 dilution). The mixtures were incubated at 37°C with vigorous shaking at 200 rpm or under static conditions. Using a spectrophotometer (Bio-Rad, USA), the OD_600_ of ZY05719 and Δ*mutT* were measured at 1 h interval for 12 h. Three independent biological replicates were measured.

### Survival assays of SS2 in H_2_O_2_, temperature, and acidic conditions

ZY05719 and Δ*mutT* were subjected to stress challenges of H_2_O_2_, high temperature, and acidic conditions, aiming to investigate the role of the deletion of *mutT* in stress tolerance. In brief, the bacteria were cultured in THB to an OD_600_ of 0.6 and collected by centrifugation at 12,000 × g for 10 min. Then, a total of 1 × 10^7^ colony-formation unit (CFU) of bacterial cells were washed twice in phosphate-buffered saline (PBS, pH 7.4) and incubated in PBS (pH 5 or 6) at 37°C for 1 h, or resuspended in PBS containing 10 mM H_2_O_2_ at 37°C for 1 h, or PBS at 40°C, 41°C or 42°C for 2 h, respectively. We determined the survival of bacteria by plating diluted samples onto THB agar in triplicate before and after stress challenges. The percent survival was determined by comparing bacterial recovery from the initial inoculum. Each assay was performed in three independent biological replicates.

### Adhesion and invasion assays

We performed adhesion and invasion assays of ZY05719 and Δ*mutT* strains to HBMEC and HEp-2 cell lines as previously mentioned [35,36]. Briefly, the wild-type and mutant strains at the mid-exponential phase were centrifuged when the OD_600_ was between 0.6–0.8. Following PBS washing twice, the pellets were suspended in DMEM infection medium. HBMEC and HEp-2 cells were grown on 24-well tissue culture plates and cultivated overnight in DMEM supplemented with 10% FBS until typically 80–90% confluence. Afterward, cells were washed in DMEM twice, followed by applying the bacteria suspension to the plates at a ratio of 10:1. The inoculated plates were then centrifuged at 800 × g for 10 min and incubated at 37°C for 2 h. After five washes with pre-cold PBS, cells were lysed by the 1% Triton X-100 for 10 min. Adhered bacteria were counted following plating serial dilutions of the lysates on plates with THB agar. Invasion assay was similarly performed to that of adhesion assay, except for applying gentamicin (100 μg/ml) and penicillin G (5 μg/ml) to kill surface and extracellular adhered bacteria. The survival percentage was determined by comparing bacterial recovery from the initial inoculum. Each assay was performed in three independent biological replicates.

### Comparative analysis and subcellular localization prediction

Complete genome sequences of available *S. suis* clinical isolates were downloaded in GenBank. The accession no. of the *S. suis* strains was provided in Table 2. Sequence alignments of the *mutT* sequence (ZY05719_09350) and the *S. suis* whole genomes were performed using the NCBI nucleotide BLAST (blastn) website (https://blast.ncbi.nlm.nih.gov/Blast.cgi). Protein subcellular localization was predicted using web servers. The amino acids of MutT were submitted to the database website Gpos-mPLoc (http://www.csbio.sjtu.edu.cn/bioinf/Gpos-multi) and PSORTb (http://www.psort.org/psortb/index.html) in FASTA format.

**Table 2.** DNA sequence analysis of the presence of *mutT* in different virulent SS2 strains

Strain	Origin	Virulence*^a^*	*mutT*	References
SC19	pig	H	+	[41]
SS2-1	pig	H	+	[42]
ZY05719	pig	H	+	[43]
SC070731	pig	H	+	[44]
S735	pig	H	+	[45]
A7	pig	H	+	[46]
GZ1	Patient	H	+	[47]
P1/7	Patient	H	+	[48]
BM407	Patient	H	+	[48]
SC84	Patient	H	+	[49]
98HAH33	Patient	H	+	[49]
05ZYH33	Patient	H	+	[20]
T15	Pig	L	-	[50]
05HAS68	Pig	L	-	[51]
90-1330	Pig	L	-	[52]
NSUI002	Pig	L	-	[53]
NSUI060	Pig	L	-	[54]

*^a^* H, high virulent; L, low virulent.

### Assessment of LD_50_ in the zebrafish model

The 50% lethal dose (LD_50_) was used to assess the virulence changes between ZY05719 and Δ*mutT* according to our previous reports [32,35,37]. Briefly, the ZY05719 and Δ*mutT* strains were cultured in THB and centrifuged at the mid-exponential phase when the OD_600_ was between 0.6–0.8. Zebrafish were anesthetized with a concentration of 90 mg/L MS-222 (tricaine methanesulfonate, Hangzhou Animal Medicine Factory) before the bacteria challenge. Zebrafish were divided to 6 groups, with 15 in each. They received intraperitoneal challenges with 20 µl of PBS containing 10^5^ CFU, 10^6^ CFU, or 10^7^ CFU of the wild-type strain ZY05719 or the ∆*mutT* strain, respectively. The bacterial CFUs were determined by plating diluted samples onto THB agar plates. Mortality was monitored daily for one-week post-infection. Infection experiments in zebrafish were performed in triplicates using the Reed and Muench method, and the LD_50_ values were calculated.

### Infection experiments in the mouse model

BALB/c mice were intraperitoneally challenged with the wild-type and ∆*mutT* strains for evaluating the role of inactivation of *mutT* on the virulence of ZY05719. Briefly, 16 female BALB/c mice with 6 weeks old were randomly divided to two groups with 8 in each. They were challenged with 200 µl of PBS containing 4 × 10^8^ CFU of ZY05719, ∆*mutT* or 200 µl of PBS as blank control, respectively. The infected mice were monitored daily for seven days. In addition, another 8 BALB/c mice were divided to two groups (n = 4) and intraperitoneally injected with a dose of 5 × 10^7^ CFU per mouse, aiming to examine the invasion and colonization capacities of ZY05719 and ∆*mutT*. At 24 h post-infection, the infected mice were sacrificed for collecting the brain, blood, spleen, and liver samples. The number of bacteria colonizing was measured by plating diluted samples onto THB agar plates.

To further compare the pathological changes in the brain of infected mice between ZY05719 and ∆*mutT*, 9 BALB/c mice were randomly divided into three groups (n = 3). Mice were intraperitoneally injected with a dose of 2 × 10^7^ CFU or PBS. At 72 h after challenge, infected mice were sacrificed for collecting brains and fixing them in 10% formalin buffer for 48 h. Brain samples were dehydrated with an automatic dehydrator, embedded in paraffin, and stained with eosin and hematoxylin for further observation under a light microscope.

### Statistical analysis

GraphPad Prism 5 software (GraphPad Software Inc., La Jolla, CA, USA) was used for statistical analysis. Differences between groups were compared by the unpaired two-tailed Student’s *t*-test. Survival rate was compared using the log-rank test. *P*-value < 0.05 considered as statistically significant. Data were expressed as mean ± SEM from three independent replicates.

## Results

### Confirmation and characterization of the mutT mutant strain

**Figure 1.**
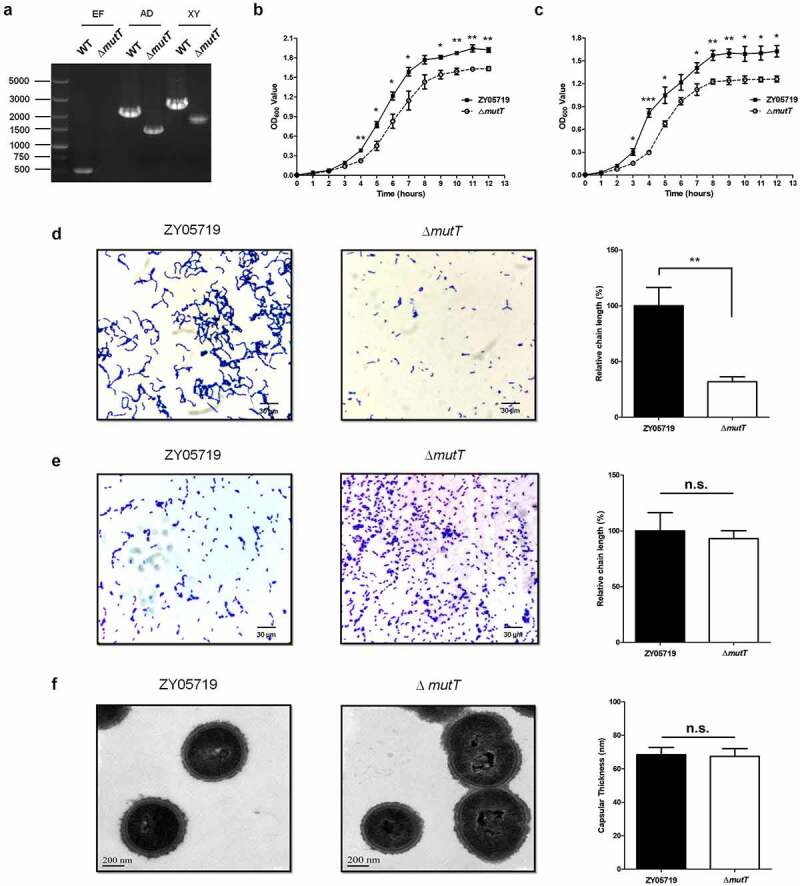
Confirmation and characterization of the *mutT* mutant strain. (a) The *mutT* mutation was confirmed through combined PCR. (b) Growth curves of the wild-type and mutant strains using OD_600_ measurements under vigorous shaking. (c) Growth curves of the wild-type and mutant strains using OD_600_ measurements under static conditions. Each experiment was performed in triplicate. *P < 0.05; **P < 0.01; ***P < 0.001. Gram staining of ZY05719 and Δ*mutT* grown in THB (d) or cultured on THA (e) were evaluated by light microscopy (× 1000). (f) Morphology characteristics of ZY05719 and Δ*mutT* were examined by TEM (× 50,000)

To examine the biological role of *mutT*, a knockout mutant of *mutT* (Δ*mutT*) was constructed by allelic replacement in SS2 strain ZY05719. We confirmed Δ*mutT* through combined PCR with three pairs of primers, followed by DNA sequencing. As shown in Figure 1(a), there were no fragments of Δ*mutT* using internal primers (E/F), and Δ*mutT* amplified smaller fragments than those of the wild-type strain using the external primers (X/Y) and flanking primers (A/D). These observations indicated that the ∆*mutT* mutant strain was successfully constructed. Then, effects of deletion of *mutT* on the growth characteristics of ZY05719 were investigated. The growth curve of Δ*mutT* in THB was significantly slower than that of the parental strain by the OD_600_ measurements under either the vigorous shaking (Figure 1(b)) or static conditions (Figure 1(c)). The Gram staining of ZY05719 and Δ*mutT* strains grown in THB were evaluated by light microscopy to investigate the influence of the *mutT* deletion. As shown in Figure 1(d), the chain of Δ*mutT* was significantly shorter than that of ZY05719 (*P* < 0.01). To avoid the accuracy of bacterial count caused by the differences of chain length, the morphologies of the ZY05719 and Δ*mutT* strains cultured on Todd Hewitt Agar (THA) were observed. There was no obvious difference in the chain length between ZY05719 and Δ*mutT* (Figure 1(e)). Therefore, in subsequent animal experiments, we cultured the ZY05719 and Δ*mutT* strains on THA. Morphology characteristics of the parent and mutant strains were further examined by TEM. There were no evident differences in morphology and capsular thickness between the ZY05719 and Δ*mutT* strains (Figure 1(F)).

### *The roles of* MutT *in oxidative stress tolerance of SS2*

**Figure 2.**
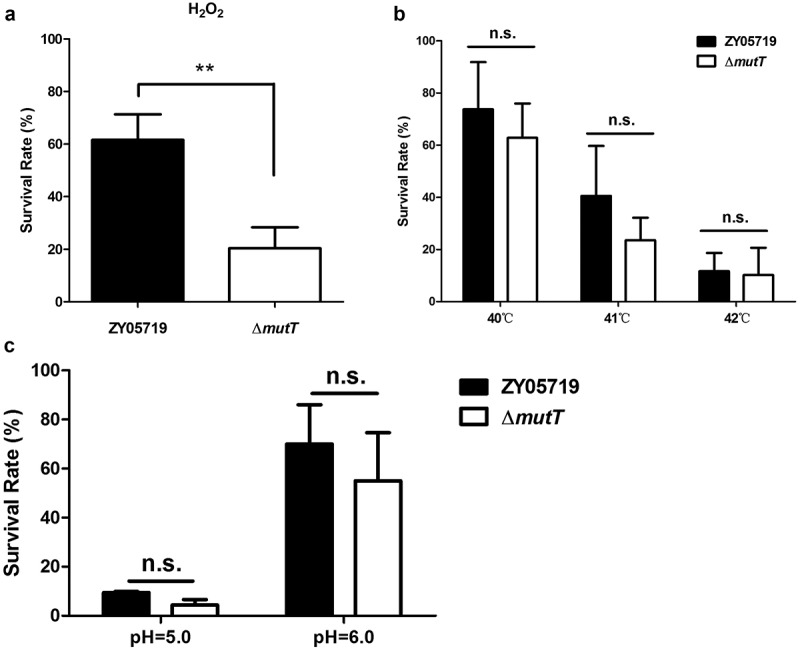
The role of MutT in oxidative stress tolerance of SS2. ZY05719 and Δ*mutT* were cultured in THB to an OD_600_ of 0.6 and collected by centrifugation. Then, the bacteria were subjected to stress challenges of H_2_O_2_ (a), high temperature (b), and acidic conditions (c). Bacterial survival was determined by plating diluted samples onto THB agar before and after stress challenges. Each experiment was performed in triplicate. *P < 0.05; **P < 0.01; ***P < 0.001

To investigate the function of deletion of *mutT* in stress tolerance, ZY05719 and Δ*mutT* were subjected to different stress challenges. We compared the survival ability of the parental and mutant strains under the stress of H_2_O_2_. As shown in Figure 2(a), the survival rate of Δ*mutT* strain was significantly lower than that of the parental strain (*P* < 0.01). However, there was no significant difference in the survival rate between the mutant and the parental strain under high temperature (Figure 2(b)) and acidic conditions (Figure 2(c)). It is concluded that MutT was of great significance in the resistance to oxidative stress.

### The involvement of MutT in adhesion and invasion of SS2 to host cells

**Figure 3.**
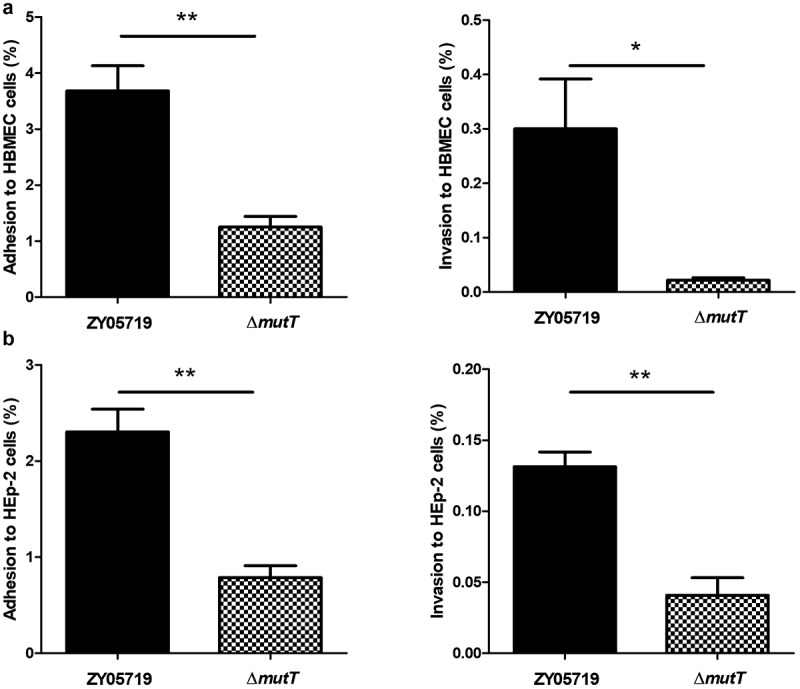
The involvement of MutT in adhesion and invasion of SS2 to host cells. (a) Adhesion and invasion of ZY05719 and Δ*mutT* to HBMEC cells. (b) Adhesion and invasion of ZY05719 and Δ*mutT* to HEp-2 cells. Each experiment was performed in triplicate. *P < 0.05; **P < 0.01; ***P < 0.001

The influence of deletion of *mutT* on the *in vitro* adhesion and invasion of SS2 was investigated using HBMEC and HEp-2 cell lines. Our results showed that the adhesion of Δ*mutT* to HBMEC cells was significantly lower than that of the parental strain (*P* < 0.01). Similarly, there was a marked reduction in Δ*mutT* invasion to HBMEC cells compared with that of the parent strain (Figure 3(a)). The adhesion and invasion rates of ZY05719 to HEp-2 cells were approximately three-fold higher than those of Δ*mutT*, which were consistent with the results in HBMEC cells (Figure 3(b)). It is suggested that MutT contributed to adhesion and invasion of SS2 to host cells.

### Colonization of the wild-type and ΔmutT strains in mouse tissues

**Figure 4.**
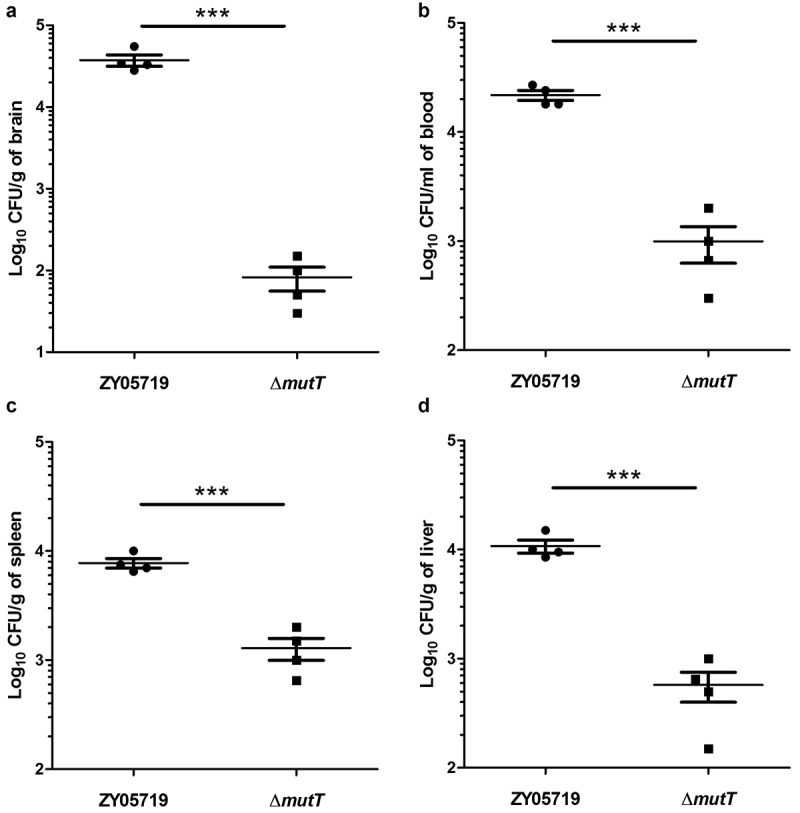
Colonization of the Δ*mutT* and wild-type strains in various tissues of mice. Mice were intraperitoneally injected with a dose of 5 × 10^7^ CFU of ZY05719 or Δ*mutT*, respectively. At 24 h post-infection, the bacteria numbers recovered in the brain (a), blood (b), spleen (c), and liver (d) samples. Results were shown as the mean ± SEM from four infected mice. *P < 0.05; **P < 0.01; ***P < 0.001

To investigate the function of *mutT* in *in vivo* infection of SS2, the colonization efficiency of the wild-type and mutant strains in BALB/c mice was compared. Mice were intraperitoneally injected with 5 × 10^7^ CFU of ZY05719 or ∆*mutT*. After 24 h of infection, infected mice were sacrificed for collecting different samples. The bacteria numbers of ZY05719 recovered from the brain (Figure 4(a)), blood (Figure 4(b)), spleen (Figure 4(c)), and liver samples (Figure 4(d)) were significantly higher than those of the mutant strain ∆*mutT* (*P* < 0.001), indicating that the *mutT* deletion mutant increased microbial clearance in host tissues of the infected mice.

### The essential role of MutT in the virulence of SS2

To investigate whether MutT was responsible for the virulence of SS2, the *mutT* sequences of 17 SS2 clinical isolates from GenBank were mined. Intriguingly, genome comparisons revealed the 17 strains of SS2 could be categorized into two groups, including *mutT*+ (12 high virulence strains) and *mutT*- (5 low virulence strains). It is shown that the presence of *mutT* was closely related to the virulence of the reported SS2 strains (Table 2). Furthermore, sequence alignment results suggested the sequence of *mutT* was 100% homology among the 12 reported high virulence SS2 strains (data not shown). Therefore, we inferred that MutT might be associated with the virulence of SS2.

We then calculated LD_50_ in parent and mutant strains in a zebrafish model, and its mean value (three replicates) was 5.02 × 10^5^ CFU and 2.08 × 10^6^ CFU for ZY05719 and ∆*mutT*, respectively (Table 3). The LD_50_ value of ZY05719 was significantly lower than that of ∆*mutT* (*P* < 0.05). In contrast, all zebrafish injected with PBS survived the entire experimental period (data not shown). This result indicated that deletion of *mutT* attenuated the virulence of SS2 in zebrafish.

**Table 3.** LD_50_ evaluation of ZY05719 and *∆mutT* with a zebrafish model

Dose of challenge (CFU)	Number of death/total					
	ZY05719			∆*mutT*		
10^7^	15/15	15/15	15/15	14/15	12/15	12/15
10^6^	11/15	11/15	8/15	4/15	5/15	4/15
10^5^	5/15	1/15	1/15	1/15	2/15	0/15
LD_50_ value	2.46 × 10^5^	5.03 × 10^5^	7.57 × 10^5^	1.63 × 10^6^	1.81 × 10^6^	2.79 × 10^6^
Mean LD_50_ value	5.02 × 10^5^			2.08 × 10^6^		

**Figure 5.**
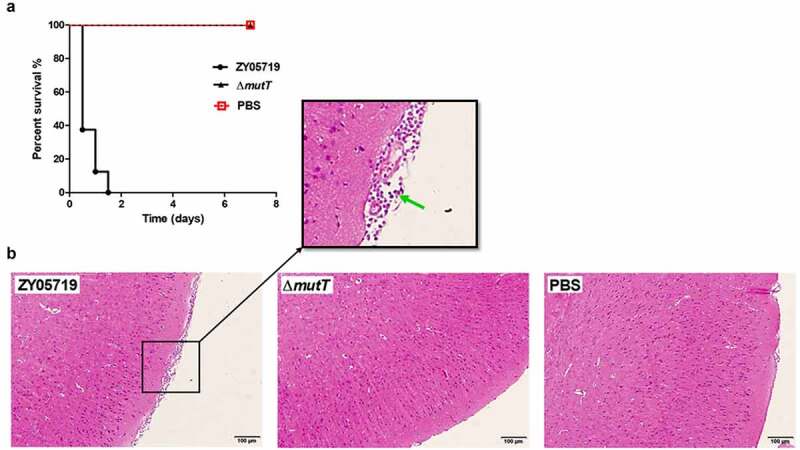
Effect of *mutT* mutation on the virulence of SS2. (a) Survival curve of ZY05719 and ∆*mutT* in a mouse infection model. Infected mice were monitored for seven days post-infection. (b) Histopathology of brain tissues of mice infected with ZY05719, Δ*mutT*, or PBS. BALB/c mice were challenged with 2 × 10^7^ CFU of ZY05719 or Δ*mutT*, respectively. At 72 h after challenge, the infected mice of each group were sacrificed for histological analysis. Brain tissue sections from ZY05719 infection group displayed meningeal thickening and neutrophilic infiltration (green arrow). No obvious pathological changes were found in ∆*mutT* group or PBS control group

Additionally, we evaluated the virulence of ZY05719 and ∆*mutT* in a mouse infection model. Mice received intraperitoneal challenges with a dose of 4 × 10^8^ CFU of ZY05719 or ∆*mutT*. In the ZY05719 infection group, all mice developed typical clinical symptoms like shivering, rough coat hair, limping, lethargy, and swollen eyes within 12 h after the SS2 challenge. All mice infected with ZY05719 died within 36 h (Figure 5(a)). In contrast, mice infected with PBS blank control and ∆*mutT* showed an overall survival rate of 100% in 7 days post-infection, and no obvious symptoms were observed. The survival rate of ZY05719 was significantly lower than that of the mutant strain ∆*mutT* (Figure 5(a)), which was consistent of that in zebrafish. It is suggested that MutT was a critical virulence factor in the pathogenicity of SS2.

Furthermore, a pathological examination was carried out to examine brain lesions in infected mice. Histological analysis of brain sections showed typical features of meningitis in the wild-type infection group, such as meningeal thickening and neutrophilic infiltration (Figure 5(b)). However, typical features of meningitis were not observed in mice of the mutant strain or the PBS control group. Collectively, these *in vivo* assays showed that MutT was essential in the virulence of SS2 in both mouse and zebrafish infection models.

## Discussion

Among all the *S. suis* serotypes, SS2 is regarded as the most important and virulent zoonotic agent, which is responsible for the infections of swine and humans. The varied virulence of SS2 strains can be attributed to their unique virulence gene profiles. Our previous pan-genome analysis identified that the gene *mutT* (ZY05719_09350) is unique to SS2 virulent strains [33]. In this study, we analyzed the *mutT* sequences of clinical isolates of available SS2 in GenBank. Our data showed that MutT consisted of 257 amino acids and the amino acid sequence of MutT was extremely conserved (100% homology, data not shown) among the 12 reported high virulence SS2 strains (SC19, SS2-1, ZY05719, SC070731, S735, A7, GZ1, P1/7, BM407, SC84, 98HAH33, and 05ZYH33). Protein subcellular localization with Gpos-mPLoc and PSORTb analyses indicated that MutT was a cytoplasmic protein. The gene *mutT* was encoded in a strain-specific manner, in which the detection was strongly related to bacterial virulence. *MutT* gene was only detected in the reported high virulence strains, but not in low virulence strains (Table 2), which was consistent with the pan-genome analysis. Therefore, we wondered whether MutT was associated with SS2 virulence. Biologic functions of MutT in SS2 pathogenicity, however, lacked further experimental evidence.

In the present work, we aim to characterize the functional definition of MutT in pathogenicity to deepen our insights of the zoonotic pathogen SS2 by investigating phenotype changes between the parental and Δ*mutT* strains. Light microscopy experiments showed that the chain length of Δ*mutT* was significantly shorter than that of ZY05719 (Figure 1(d)). Rodriguez et al. reported that the long-chain enhances the adhesion ability of *S. pneumoniae* to human epithelial cells *in vitro* and colonization in a murine model *in vivo* [38]. Long-chain formed by deletion of SS2 *msmK* stimulates adhesion to HEp-2 cells [39]. Very recently, Liu et al. showed that inactivation of SS2 *prsA* increases the chain length and enhances adhesion to host epithelial cells [40]. In the present study, two different cell lines (HBMEC and HEp-2) were chosen to test the contributions of deletion of *mutT* on the adhesion and invasion by SS2. It is found that the *mutT* deletion mutant significantly inhibited adhesion and invasion of SS2 to host cells (Figure 3), which may be partially explained by the reduced chain length of ∆*mutT*. Adhesion is a critical step to initiate the bacterial invasion process, and it was a potential reason for the attenuated invasion of SS2 to host cells by deletion of *mutT*.

To establish a successful infection model, SS2 must overcome adverse environmental conditions, such as oxidative stress, high temperature, and acidic conditions. We examined the characteristics of ∆*mutT* in stress tolerance. As shown in Figure 2(a), the survival rate of Δ*mutT* strain in a microenvironment of H_2_O_2_-induced oxidative stress was largely reduced in comparison to that of the wild-type strain. Consistent with the findings by Tan et al. [39], the reduced tolerance of ∆*mutT* to oxidative stress might be an important factor for the reduced survival of the mutant in the infected mice tissues (Figure 4), because ∆*mutT* was probably less adapted to the host environment during infection.

To better characterize the function of MutT in *in vivo* infection of SS2, we compared the virulence of ZY05719 and ∆*mutT* in a zebrafish infection model. LD_50_ value of ZY05719 was much lower than that of ∆*mutT*, indicating that deletion of *mutT* attenuated the virulence of SS2 in zebrafish. Bacterial invasion and colonization capacities in host tissues and bloodstream are considered as critical events in SS2 pathogenesis [21]. We established an *in vivo* mouse model to investigate biological role of MutT in SS2. As expected, the number of viable bacteria of ∆*mutT* recovered from the brain, blood, spleen and liver samples was significantly lower than those of ZY05719 (Figure 4). Further *in vivo* assays showed that deletion of *mutT* significantly inhibited the viability (Figure 5(a)) and histopathological lesions (Figure 5(b)) in a SS2 infection model in BALB/c mice. Taken together, MutT was essential for the full virulence of the zoonotic pathogen SS2 in both mouse and zebrafish infection models. Notably, the standardization of animal models for SS2 infection is still controversial. The mouse and zebrafish infection models have many advantages like simple procedures, low cost and large sample size, limitations remain to be solved. Dominguez-Punaro et al. showed that mice can develop typical clinical symptoms of *S. suis* disease, including septicemia, meningitis and septic shock followed by clinical signs of central nervous system (CNS). CNS clinical symptoms are difficult to be evaluated, which is the major limitation of zebrafish infection model. In conclusion, some results obtained from mouse and zebrafish studies of *S. suis* disease might not be extrapolated to the natural host of pigs.

In summary, our study identified a novel virulence factor MutT in the representative Chinese virulent isolates of zoonotic pathogen SS2. The present study confirmed that MutT is required for the full virulence of SS2. Our results provide useful insights into the biological functions of MutT in the pathogenesis of *S. suis* infection.

## Data Availability

Data sharing is not applicable to this article as no new data were created or analyzed in this study.
